# Relief Modeling in the Restoration of Extractive Activities Using Drone Imagery

**DOI:** 10.3390/s23042097

**Published:** 2023-02-13

**Authors:** Erick Russell, Joan-Cristian Padró, Pau Montero, Cristina Domingo-Marimon, Vicenç Carabassa

**Affiliations:** 1 Universitat Autònoma de Barcelona, E08193 Bellaterra (Cerdanyola del Vallès), Catalonia, Spain; 2 Departament de Geografía, Edifici B, Universitat Autònoma de Barcelona, E08193 Bellaterra (Cerdanyola del Vallès), Catalonia, Spain; 3 CREAF, E08193 Bellaterra (Cerdanyola del Vallès), Catalonia, Spain; 4 Grumets Research Group, CREAF, Edifici C, Universitat Autònoma de Barcelona, E08193 Bellaterra (Cerdanyola del Vallès), Catalonia, Spain

**Keywords:** mines, drones, photogrammetry, interpolation, modeling, DEM, water flow, visual basin

## Abstract

In the field of mine engineering, a cross-section topographic survey is usually carried out to perform volumetric calculations of earth movement in order to restore areas affected by extractive activities. Nowadays, Remote Sensing and Geographical Information System (GIS) technologies make it possible to perform the same work by using indirect methods such as images obtained by photogrammetric flights. In this context, Unmanned Aerial Systems (UAS) are considered a very convenient option to develop mapping projects in short periods of time and to provide quality geospatial information such as Digital Elevation Models (DEM) and orthophotos of centimetric spatial resolution. In the present study, this approach has been applied in a gravel extraction area to obtain data for estimating the filling volume of material required for the restoration of the relief (DEM(r)). The estimation of the DEM(r) is later used to calculate a difference of height values (DEM(r)-DEM) that will serve as a variable in the basic operation of volume calculation. The novelty of the presented method is the simulation of a relief adapted to the surrounding morphology, including the derived channel network and the visibility impact, improving what would be a simple clogging. Likewise, the generation of 3D models allows visualizing a new morphological structure of the relief. The proposed approach, based on GIS tools, allows analyzing water flow connectivity integration of the DEM(r) with the environment and estimating potential landscape impacts from the main focuses of a visual basin, both of which are key aspects of restoration modeling that are not always properly addressed.

## 1. Introduction

Mining activities are very important for the provision of raw materials and energy [1]. However, this activity generates changes in the morphology and structure of the landscape, altering the natural conditions of the area and directly and indirectly influencing the conditions of its closest environment. It has environmental impacts, such as loss and pollution of soils, vegetation cover, erosion, unstable slopes, detachments of loose material, and alteration of the flow of groundwater and surface water, among others [2].

These impacts are the reason why organizations such as the United Nations recommend improving the management of environmental impacts and, thus, complying with Sustainable Development Goal 12 of Responsible Production and Consumption [3]. In this sense, Spanish legislation requires companies to implement restoration plans that apply corrective measures to prevent, reduce, and rehabilitate the land affected by the extractive activity. These plans aim to return the land to a satisfactory state to the greatest degree possible, guaranteeing a balance of the natural space before the exploitation operations (Royal Decree 975/2009). A restoration plan should involve the recovery of environmental functions and services lost during the exploitation phase, such as the regulation of the hydrological cycle, the recycling of organic matter, and the production of biomass or biodiversity so that we can talk about ecological restoration [4]. Beyond the work of creating new soils or planting forest species, the restoration of these functions depends in great measure on the correct modeling of the terrain [5]. In this context, the calculation of the filling volume necessary for correctly modeling the terrain has been addressed by topographic surveys in the field (direct methods), which are used to learn the physical characteristics of the land surface. The topographic surveys result in vector entities of type point (height levels) or lines (contour lines) that are used to generate triangulation models, such as the Triangulated Irregular Network (TIN). This model presents a simple structure based on the connection of vertices of the nearest neighbors to form triangular networks, obtaining a representation of the morphology of the terrain [6]. TIN models are commonly used in engineering work to calculate volumes from cross-sections because it is an easy method to understand and implement. However, its results are usually not accurate as it assumes that the cross-sectional area varies linearly with the length [7]. Other interpolation methods, such as Inverse Distance Weighting (IDW) [8], splines [9], or kriging [10] are used for mineral resources. TIN, IDW, splines, and kriging interpolation tools [6,11,12] are available in common GIS software (MiraMon [13], QGIS [14], ArcMap [15]) toolbox obtaining reasonable results in this and previous studies [16,17].

Nowadays, there are other mechanisms and techniques that allow studying the changes generated in the territory due to mining activity with higher precision than interpolating classical topographic surveys. For instance, spatial remote sensing offers numerous advantages, complementing other conventional means of observation. One of the most outstanding contributions is its ability to follow dynamic processes, making it an invaluable source for studying changes that occur on the earth’s surface [18]. On the other hand, the accelerated development of technology has allowed the refining of small platforms that integrate airborne optical sensors in order to capture remote information in short periods of time, with good spatial resolution and low cost [1]. This technology has already been applied to quarry explorations [19]. Similarly, Unmanned Aerial Systems (UAS) have become a useful source for monitoring and analyzing morphometric changes generated by extractive activities through detailed photogrammetric flights, from which orthophotos and Digital Elevation Models (DEMs) of centimeter-level resolution are obtained [20]. In this case, the variation of extracted volume can be determined through the detection of very accurate changes in altitude between the current state of the terrain (once the extractive activity has ended) and the original state (prior to the start of mining). In the absence of a DEM prior to the start of mining, a possible solution is to make an estimate based on the interpolation of height references of unaltered locations during the mining activity, as has been done in erosion studies [16,17]. The identification and digitalization of these points are performed by expert visual interpretation supported by the use of orthophotos. The corresponding altitude value is added as an attribute according to the DEM generated. However, some of those methods are more complex to use and not applicable to steep slopes like those that are dominant in the vast majority of the restorations carried out in the context of open mining.

The main objective of this study is to propose an approach to estimate a relief model that simulates the filling surface (Digital Elevation Model of the filling volume of material (DEM(r))). This model allows an approximate quantification of the volume required to restore the gravel pit based on the UAS-extracted terrain model and point interpolation techniques from witnesses’ points that try to reproduce the original morphology in order to have a better approximation of the volume to be filled. The main novelty of the proposed methodology is to analyze the integration of this filling model with the morphological structure of its environment, focusing on the connection with the water network and subsequent analysis of the impact of the visual basin. A secondary objective is to provide a GIS tool to the readership capable of reproducing the methodology described (in this case, implemented in ArcMap 10.7).

## 2. Study Area, Materials, and Methods

### 2.1. Study Area

**Figure 1:**
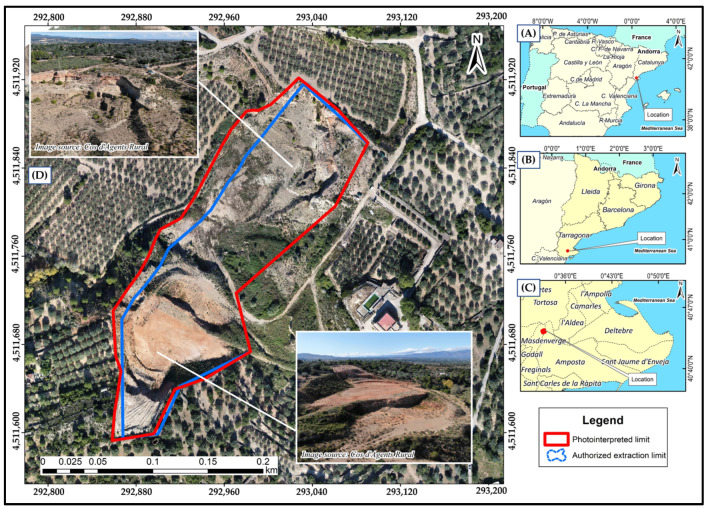
(**A**) Location of the study area at the autonomous precinct level. (**B**) Location at the province level. (**C**) Location at the municipality level. (**D**) Orthophoto view of the Belen waste dump.

The study area is located in the region of Montsià, municipality of Masdenverge, province of Tarragona—Catalonia (Northeast of Spain) (Figure 1A,B). The extractive activity called Belén EA20040002 is located at the geographic coordinates Latitude 40°43′49″ N and Longitude 0°32′54″ E, between 53 and 87 *m.a.s.l*. The company Construcciones y Ayudas Ferroviarias, S.L. dedicated to the extraction of gravel is operating it [21].

The official boundaries of the Belén gravel pit were downloaded from the Hipermapa viewer, the catalog of layers of extractive activities of the Government of Catalonia [21]. According to the official boundaries, the extraction area is 2.71 ha, which is replaced by a larger area of 3.24 ha after assessment by photo-interpretation (see Section 2.3.1) (Figure 1).

**Figure 2:**
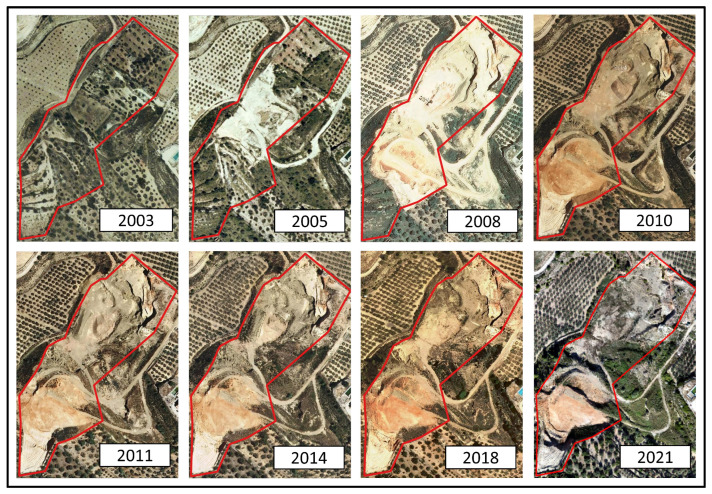
Multitemporal aerial orthophotos of the period 2000–2021 [23].

At the geological level, the substrate is generally composed of sedimentary material and rocks (grayish limestones, gray, black and red shales, and blackish peats) [22]. According to the visual analysis of the time series 2000–2021 orthophotos [23], it can be inferred that the exploitation activities began between 2003 and 2004 because in previous years no outstanding change of earthwork in the study area was visualized. In contrast, 2005 images show very characteristic bright tones of bare ground and the establishment of access for heavy vehicles. From that point on, the extraction of material modified the natural environment, mainly the topography and the vegetation cover, initially in the central and northern sectors. In 2010, the entire authorized area was already exploited and during 2011, the extraction operations at the central and northern sectors finished, resulting in the growth of small bushes and grasslands. In 2021, the whole area was already undergoing a process of abandonment, pending the start of restoration actions (Figure 2).

### 2.2. Materials

#### 2.2.1. Drone Data

A DJI Phantom 4 Pro drone multicopter (four-rotor) with an RGB 1″ CMOS sensor (DJI) [24] has been used for capturing images by planning and executing a photogrammetric flight (Table 1). The Rural Agents of Catalonia carried out the photogrammetric flight on 25 October 2021, at 1 pm (CEST) approximately. The flight altitude was 100 m, with a frontal overlap of 80% and a side lap of 70%, obtaining a pixel spatial resolution of 5 cm. After photogrammetric and SfM software post-processing (see Materials section), a DSM (Digital Surface Model), a DEM, and an orthophoto were obtained.

#### 2.2.2. Lidar Data

Lidar (Laser Imaging Detection and Ranging) data were downloaded from the official mapping local agency, Institut Cartogràfic i Geològic de Catalunya [25]. It corresponds to the second coverage of Catalonia carried out in 2016. Lidar points are distributed in LAZ/LAS format by blocks of 2 km × 2 km which guarantees that 95% of the block area has a minimum density of 0.5 points/m^2^, resulting in a DEM of 2 m pixel size (DEM-LIDAR). The six available quadrants of Lidar data were mosaicked, and noise filtering was applied by eliminating values of noise categories 1, 7, 11, and 13 according to the table of categories and basic description of LAZ files [25,26]. The interpolation of Lidar points was performed using the neighborhood statistics method, in which the mean value of all the points that are within a radius of 2 m is assigned to each cell. Likewise, in the interpolation tool, a selection of classes 2 and 8 (ground points) was applied, in order to eliminate higher values of ground level. A 2 m × 2 m pixel size DEM with a coverage of 12 km × 12 km was obtained, which covered the surrounding area of the gravel pit. Lidar point cloud data were processed using LAStools [27] software. For the GIS analysis as well as for the modeling, ArcMap 10.7 and MiraMon software have been used.

### 2.3. Methods

**Figure 3:**
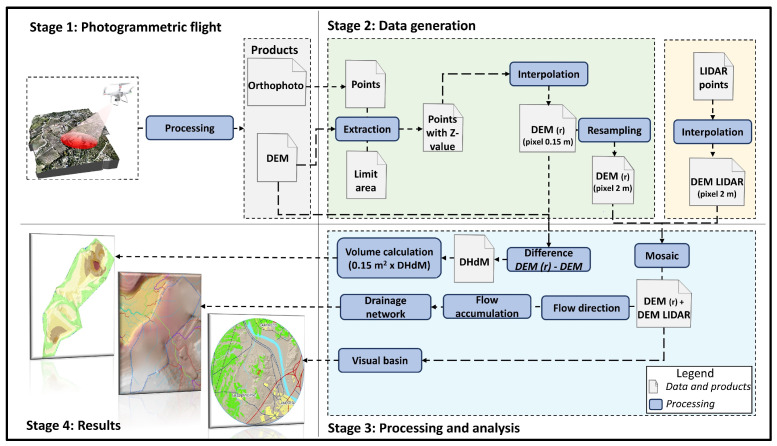
Methodology workflow, from Stage 1 (photogrammetric flight), Stage 2 (Data generation), Stage 3 (Processing and analysis), and Stage 4 (digital mapping results).

The methodology applied to estimate a restored filling model that serves to perform volume calculations and analyze its integration into the environment through the analysis of water flow and its impact on the visual basin is structured in 4 stages: (1) Photogrammetric flight, (2) Data generation, (3) Processing and analysis, and (4) Presentation of results (Figure 3). Each stage is described in detail hereafter.

#### 2.3.1. Photogrammetric Flight

Agisoft PhotoScan [28] photogrammetric and Structure from Motion (SfM) software has been used to process the flight data, obtaining a Digital Surface Model (DSM) and a Digital Elevation Model (DEM) of 15 cm spatial resolution as well as an orthophoto of 5 cm spatial resolution. For the drone-derived product georeferencing, corresponding coordinates between the drone images and the official mapping agency images [25] were used as control points; this allowed an accurate fitting of the drone orthophoto and the official agency orthophoto, and also between the drone-derived DEM (DEM-DRONE) and the official agency DEM [16]. This georeferencing method is crucial for further matching between the DEM-DRONE and the surrounding DEM-LIDAR. The imagery post-processing workflow used is defined in several previous studies carried out by the authors [29,30,31,32].

#### 2.3.2. Data Processing and Analysis with GIS (I): Exploited Area Boundaries vs. Official Boundaries

The first analysis required for an extractive activity restoration is to evidence if the real exploited area is located inside the area conceded to extraction. Boundaries of the real exploited area were photo-interpreted performing a manual digitization of the contour of the area that was truly affected using the drone orthophoto. The result was inconsistent with the official limits of the gravel pit, the latter being much smaller. Therefore, a new demarcation was defined in order to achieve a better representation of the relief of the gravel pit. This matching analysis is essential for quantifying the real affected area to restore, and for environmental administration.

#### 2.3.3. Data Processing and Analysis with GIS (II): Generation and Validation of a Restoration Relief Model (DEM(r))

Often, there is no available cartographic information on the study area before the beginning of the exploitation phase. This was the case with the Belén gravel. Therefore, it was necessary to estimate a DEM for this specific momentum, called time 0 (T0), to model the projected restored terrain DEM(r). The strategy assumes that the borderline of the extractive basin is the only altitude reference from the past. The reference values for generating the DEM(r) were extracted from the overlap of the contour of the gravel pit with DEM-DRONE resulting from the drone photogrammetric flight. Given that the drone flight is georeferenced using control points extracted from the DEM-LIDAR data, the method assures the integration of DEM(r) through the contour line interface, with the immediate surroundings represented by the DEM-LIDAR. This way, the filling model was spliced with the current configuration of the adjacent relief, thus avoiding abrupt changes in slope.

A continuous surface DEM(r) in a grid format of 15 cm resolution was generated by interpolating z-coordinates (altitude) located on the dividing ridge of a basin. However, as in the DEM-LIDAR, the interpolation of the points was also performed using the neighborhood statistics method, obtaining a 2 m pixel size grid.

**Figure 4:**
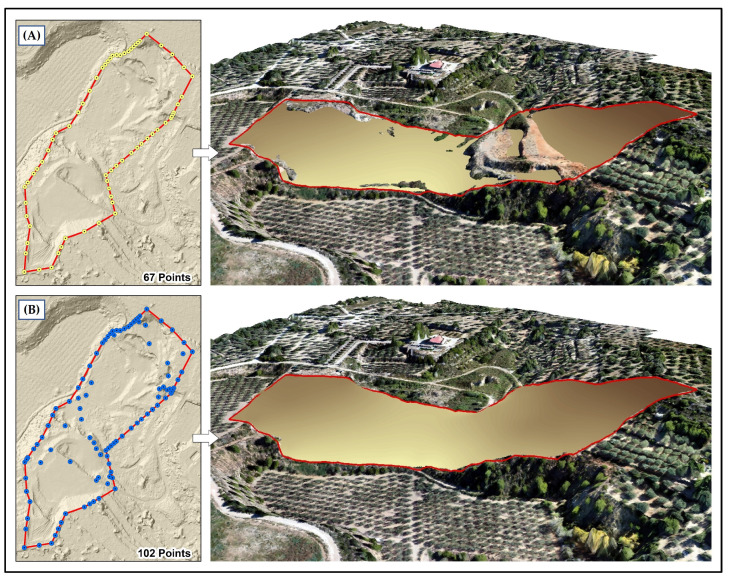
DEM(r) generated from (**A**) interpolated points only at the gravel limit and (**B**) adding points at maximum altitude locations inside the gravel pit.

The DEM of the restored relief (DEM(r)) obtained as a first result is a model that simulates the filling surface required. However, the authors advise that it can present some imperfections (Figure 4A) because only altitude values corresponding to the contour of the gravel pit were initially chosen, without considering higher altitude values within the area. The lack of references inside the area can generate a filling model presenting protrusions corresponding to the higher altitude values. In order to improve the final relief and the restoration model of the morphology of the land, the method includes new points that have been added at the maximum height locations within the gravel pit (in this study case 35 new points) based on the higher peak values of the actual drone derived DEM-DRONE (Figure 4).

The integration of the DEM(r) with its environment was made by merging this DEM(r) with the DEM-LIDAR. It is worth noting a key step to success in this integration: the control points to georeference of the drone photogrammetric flight were obtained from the information based on the Lidar data; therefore, there is no step effect between both DEMs. To complete the integration of the DEM(r) and DEM-LIDAR, the rasters were adapted to the same grid at 2 m pixel size, and a mosaic was performed to integrate both files (Figure S1). For practical purposes, this result was called the Integrated Digital Elevation Model (DEM(i)).

#### 2.3.4. Data Processing and Analysis with GIS (III): Total Filling Volumetric Calculation

To perform the volumetric calculation of filling required to restore the gravel pit, the classic volume formula (1) was applied at the pixel level. A final sum of all the values obtained will be performed to compute the total volume of filling.

*V* = *Σ*(*a*^2^ × *h*) (1)

where *V* is the volume, a is the pixel resolution (15 cm or 255 cm^2^) and *h* is the Digital Height Difference Model (DHdM) [31], obtained from the difference between DEM(r) and DEM-DRONE.

**Figure 5:**
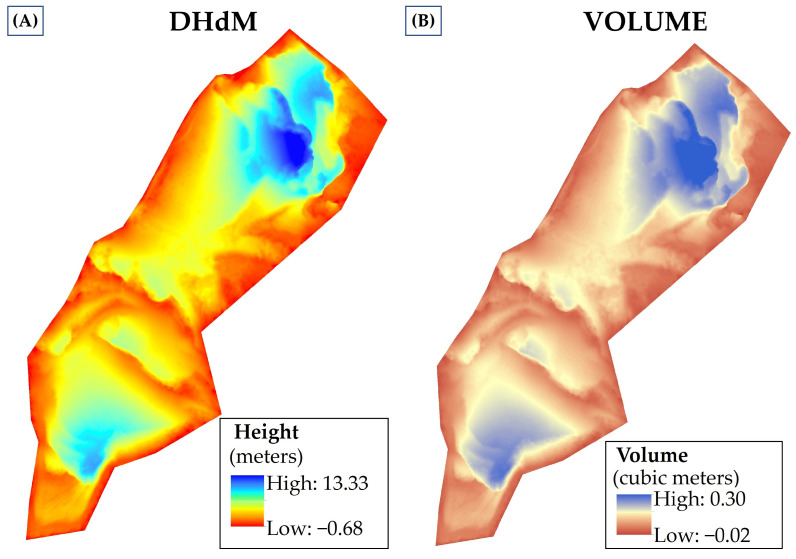
(**A**) Calculation of the height difference (DHdM) value between the restored DEM (DEM(r) and the drone-acquired DEM-DRONE. (**B**) Calculation of the volume needed to obtain the DEM(r), obtained by multiplying the pixel area (in this study case 0.255 m^2^) and the DHdM pixel value.

The operation was performed on a raster calculator that operates at the pixel level, resulting in a new spatially detailed map informing about the height attribute (*h*) required to reach the fill level at each cell. This attribute is then transformed to the volume of filling (m^3^) required per pixel to restore the morphology of the terrain by multiplying each pixel attribute by its own area (in this study case, at 15 cm × 15 cm the pixel area is 0.0255 m^2^) (Figure 5 and Figure S1). A total volume of filling is obtained by adding up the values of each pixel.

#### 2.3.5. Data Processing and Analysis with GIS (IV): Water Flow Modeling

This application is specifically designed for the creation of hydrological modeling, taking into account the maximum and minimum levels of the DEM(i) input file, generating a model that simulates a drainage structure without losing the surface continuity of the relief.

To analyze the integration of the estimated filling model with its environment, it is considered appropriate to simulate the spatial distribution of the surface runoff of the area to be restored, understanding that the runoff is conditioned by the morphology of the terrain.

The direction of the decline of each cell is calculated through the altitude values of DEM(i), taking as a reference the method of D8 [32], which considers that a cell has the option of the direction of its 8 closest neighbors, choosing the one with the lowest value. According to the number of times a cell converges towards another, a weight value is assigned, and the cells with the highest number of flow accumulation (higher weight) are classified as channels of the water flow network. At the scale of this study case, cells with an accumulated flow weight value > 50 were considered water stream channels; cells with values < 50 were assigned null values; cells of weight 0 identified the ridges (see Figure S2 in Supplementary Materials).

#### 2.3.6. Data Processing and Analysis with GIS (V): Visual Basin Modeling

In order to analyze the landscape impact generated by the restored relief of an extractive activity, it is important to evaluate the visual scope that is perceived from different observation points. The calculation of the visual basin was based on DEM(i). Points of maximum height levels within the limit of the gravel pit were classified as observation points, taking a buffer of 4 km and considering elements such as roads and urban centers. ArcMap 10.7 visibility tool was used to calculate the visual basin [33], which determines the raster surface locations visible to a set of observer features or identifies which observer points are visible from each raster surface location. The result is a binary map assigning values 1 to visible cells and values 0 to non-visible cells.

## 3. Results

The first result derived from the drone imagery is the identification of discrepancies between the real exploited area boundaries and the official boundaries. This is due to the extraordinary detail of the drone imagery lower than 10 cm, which allows for a very accurate photo-interpretation. The second important result is obtaining a restoration relief modeling (DEM(r)) based on the DEM-DRONE heights of the exploitation contour boundaries and peak point heights inside the exploitation basin. This result allows the calculation of a volumetric estimation for the filling restoration. A third result is obtaining the integrated DEM(i) based on the merging of the DEM(r) with the official mapping agency DEM (DEM-LIDAR). This result allows the calculation of the resulting hydrological channel network and the visual impact. Let us look at each result in detail.

### 3.1. Volumetric Calculation

**Figure 6:**
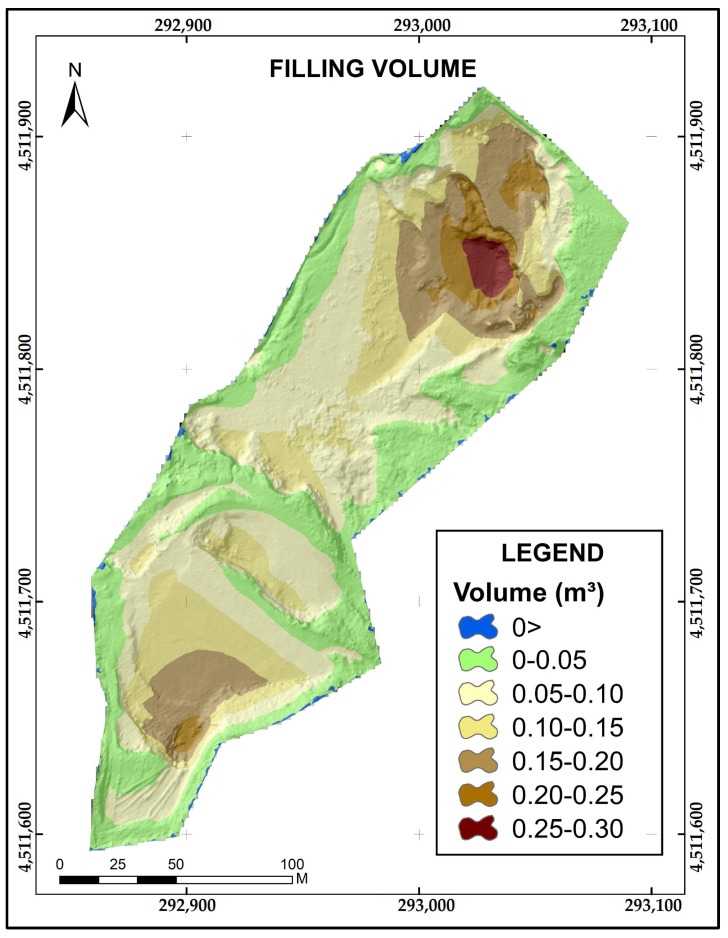
Spatial distribution of the volume required to restore the Belén gravel pit.

The quantitative analysis of the inner extractive basin is important for both the proprietary benefits and the environmental restoration. The volumetric calculation, computed as the difference (DHdM) between DEM(r) and DEM-DRONE, gives a 15 cm × 15 cm sampling grid for the entire pit area. Each pixel area, multiplied by the height difference value, offers extremely detailed volumetric data in spatial terms. A total of 1,428,467 pixels of 15 cm × 15 cm were classified as filling pixels and as a function of the filling volume in order to identify the severity of the restoration needs (see Figure S3). The total volume required to restore the relief of the Belen gravel pit corresponds to 124,941 m^3^. The area and volume required to restore the gravel pit are distributed as shown (Table 2 and Figure 6).

**Figure 7:**
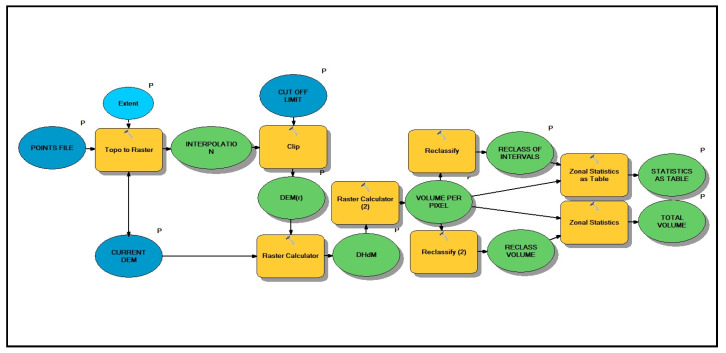
Volume calculation workflow diagram in ArcMap 10.7. The reader can find the tool in the Supplementary Materials (Volumetric_Tool.zip).

It is important to present the GIS tool that automatizes the generation of the relief restored DEM(r) and compares it with the current relief DEM, obtaining a raster of differences (DHdM) and a statistics report (Figure 7 and Supplementary Materials).

### 3.2. Water Flow Modeling Results

A very dense water network has been obtained, not only within the restored model but also integrated into the surrounding relief.

**Figure 8:**
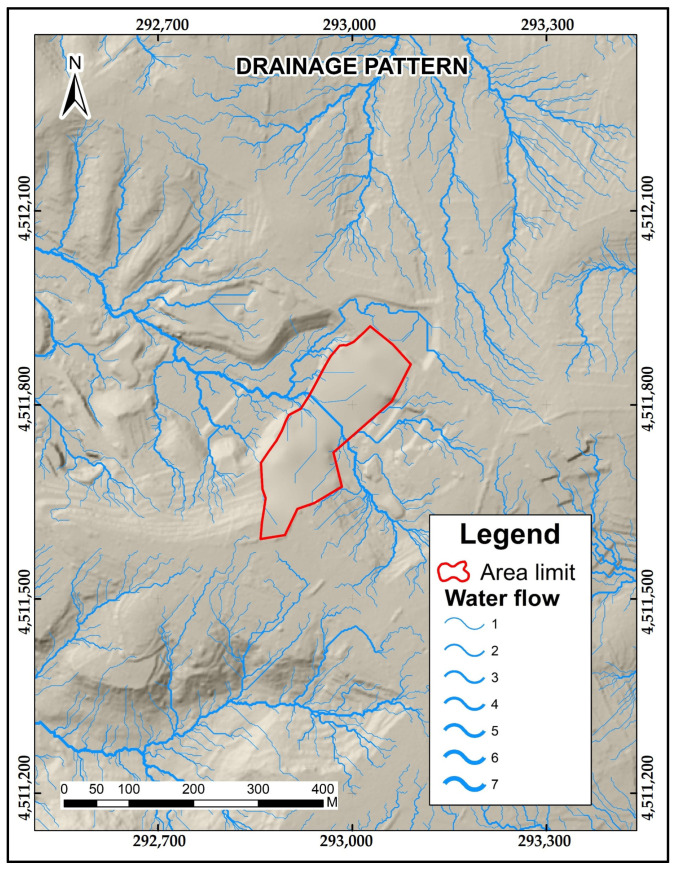
Drainage pattern integrated into the environment.

The method allows observing that the estimated relief restored model presents good results since the water flow is not interrupted and/or because the flows generated by the morphology follow a course and are integrated into the main network (Figure 8).

### 3.3. Visual Basin Modeling Results

**Figure 9:**
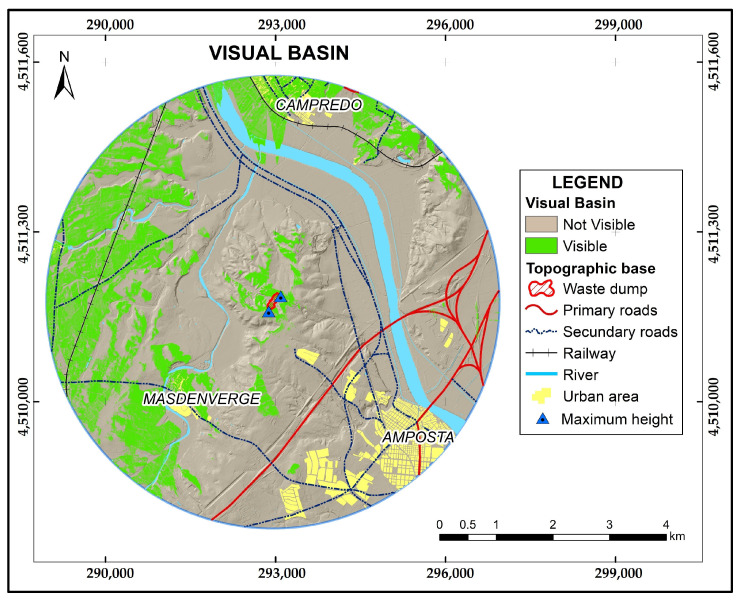
Visual basin with a radius of 4 km generated from two maximum points identified at the northern (76 m) and southern (88 m) ends.

Results of the analysis of the visual basin of the gravel pit indicate that it has a visual impact of 17% on areas located within the 4 km buffer, so 83% of the watershed has no visual contact with the gravel pit. Two urban centers (Campredo and Masdenverge) have also been identified within the impacted area, as well as two secondary roads and a railroad (Figure 9 and Table 3).

## 4. Discussion

Drones offer very good quality products, such as centimeter-level resolution DEMs, which are very useful for terrain morphology studies. At the same time, it is important to have time series data, since from this information, it is possible to analyze dynamic processes such as changes and land use in the territory [34,35]. Specifically, in applications oriented toward the restoration of extraction areas, it could be considered a disadvantage not to have data prior to the start of operations in the gravel pit, since information regarding the physical conditions of the territory is lost. To overcome this lack of information, it is possible to estimate the relief morphology using terrain references and interpolation methods [16,17].

The methodology proposed in this study considers generating a simple relief model estimated from elevations obtained from the contour of the extraction area as a basic proxy for the initial estimation of filling volume in geomorphological restoration. However, the method overpasses the problem of inner elevations in the gavel basin. When superposing the DEM(r) with the DEM-DRONE (current state of the relief), some elevations higher than the estimated model were identified, due to the existence of altitude values higher than the filling model. The analysis of this group of values revealed that they correspond to slightly altered areas that maintain topographic conditions of the original relief, so it is essential to identify these maximum elevations and add them to the interpolation method in order to estimate a filling model that completely covers the exploited area. The application of these fills can ensure the morphological integration of the gravel pit to its immediate surroundings, reducing external dynamic effects such as erosion, landslide, and rock fall, among others [36]. The volumetric calculation by indirect methods could have better results than those obtained through conventional methods, since the proposed methodology works with information at a pixel level of 15 cm spatial resolution, compared with the volume calculation method by cross sections that averages contiguous areas [7].

The integration of the modeled relief restored DEM(r) with the official mapping agency surrounding DEM (DEM-LIDAR) is crucial for the correct analysis. The integrated DEM (DEM(i)) was assured by georeferencing the drone flight using control point coordinates obtained from the DEM-LIDAR, and thus, the merging of both DEMs was good enough to avoid errors in the hydrological analysis. If this integration is wrong, it may present errors at the extremes, over the contour area, showing step pattern effects in the channel network. As an alternative to photo-interpreted control points from the official maps, in situ topographic work can improve the delimitation of the study area and capture altimetry data of time-invariant points to refine the interpolation model and obtain a finer integration of the filling model.

The detection of water flow is an element of analysis that corroborates the integration of the model in the landscape; it also confirms that the estimated filling model is morphometrically close to the desired relief for restoring the gravel pit [31]. The water runoff path identification and the Strahler order [37] of each channel point out the main water erosion threat locations and allow tracking of the lixiviated particles.

Finally, the analysis of the visual basin has shown that the integration of the UAS-extracted terrain model with the Lidar data can quantify, with high accuracy, the potential areas with views over the open pit mine. This is therefore a useful, fast, and accurate tool for decision making regarding the visual impact generated by extractive activities.

## 5. Conclusions

Geomorphological restoration modeling of extractive activities has multiple solutions. This paper presents an approach based on the calculation of the volume of filling material needed for the restoration, based on information from drones and GIS processing. Nevertheless, the main contribution is the expansion of the scope out of the exploited area by analyzing the integration of the relief restoration model with its surrounding relief. The clue of the method resides in georeferencing the drone-derived Digital Elevation Model using photo-interpreted control points obtained from the official agency Digital Elevation Model, thus assuring a fine merging between both. Results show that the method improves the relief modeling of the restored space and, especially, contributes to the combined study of the modeled relief and the current relief context. Likewise, the modeled relief integration with the environment is corroborated by analyzing the water flow behavior. Although this method is still under users’ evaluation (users can test the provided tool (see Supplementary Materials)), this approximation to the final morphology is valid for estimating in an easy and very precise way the amount of filling material necessary to restore the morphology of the extraction pit and for modeling of its consequences to the environment. The method contributes to decision-making criteria for the treatment and morphological restoration of these types of spaces since the information has a very good spatial resolution, and the mathematical operations are carried out at a very high-resolution pixel level. Further research on the analysis using more variables to guarantee its effectiveness is needed, which will add more certainties to the models.

## Figures and Tables

**Table 1:** Characteristics of the drone used (Phantom 4 Pro) [24].

Width	5472 px	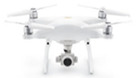
High	3648 px	
Resolution	20 mpx	
Bands	R,G,B	
Focal	f/2.8–11	

**Table 2:** Volumetric calculation by the intensity of volume class.

Classes m^3^	Area m^2^		Volume m^3^	
0	209.97	209.97	0.00	0.00
0–0.05	10,401.93	Total landfill area 32,140.49	12,546.50	Total backfill volume 124,941.22
0.05–0.10	10,391.96		34,645.90	
0.10–0.15	5862.53		31,384.08	
0.15–0.20	3857.92		29,642.78	
0.20–0.25	1226.48		11,887.03	
0.25–0.30	399.67		4834.93	

**Table 3:** Results obtained from the visual basin.

Type	Area (m^2^)	Percentage (%)
Visible	8,642,592 m^2^	17
Not visible	41,621,176 m^2^	83
Total	50,263,768 m^2^	100

## Data Availability

All drone and airborne orthophotos, DEMs, shapefiles and code will be made available on request to the correspondent author’s email with appropriate justification.

## Supplementary Materials

The following supporting information can be downloaded at: https://www.mdpi.com/article/10.3390/s23042097/s1, Figure S1: Integration of DEM-LIDAR and DEM (r); Figure S2: Reclassified raster with flow accumulation values > 50 and null values; Figure S3: Classification of raster in cut and filling categories. The following file was also added: Volumetric_Tool.zip with the ArcMap 10.7 tool to calculate the restored DEM with sample data.

## Abbreviations

The following abbreviations are used in this manuscript:

DEM	Digital Elevation Model
DEM(i)	Integrated Digital Elevation Model
DEM(r)	Digital Elevation Model of the filling volume of material (restoration relief)
DEM-DRONE	Digital Elevation Model derived from drone
DEM-LIDAR	Digital Elevation Model derived from lidar
DHdM	Digital Height Difference Model [DEM(r)-DEM-DRONE]
DSM	Digital Surface Model
GIS	Geographical Information Systems
IDW	Inverse Weighted Distance
SfM	Structure from Motion software
TIN	Triangulated Irregular Network
UAS	Unmanned Aerial Systems
