# Supplementary material for: Relief Modeling in the Restoration of Extractive Activities Using Drone Imagery
Source: Sensors (Basel). 2023 Feb 13;23(4):2097. doi: 10.3390/s23042097 (PMC9963660; doi:10.3390/s23042097)
Supplement: Supplementary file 1 [file sensors-23-02097-s001.zip › 05_Russell_Sup_Mat_v5.pdf]

sensors

Supplementary Materials

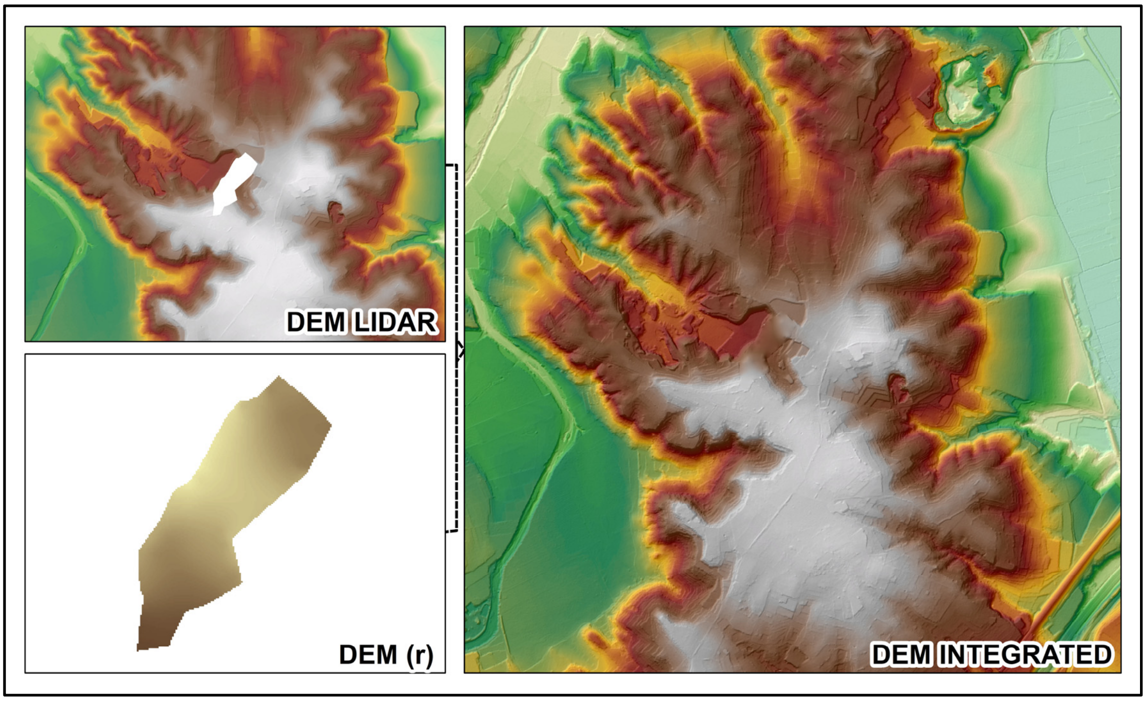

Figure S1. Integration of DEM-LIDAR and DEM (r).

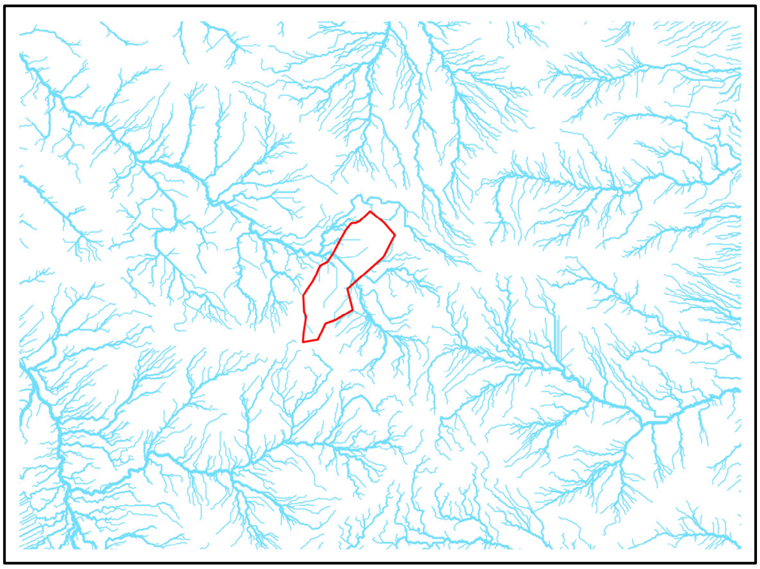

Figure S2. Reclassified raster with flow accumulation values > 50 and null values.

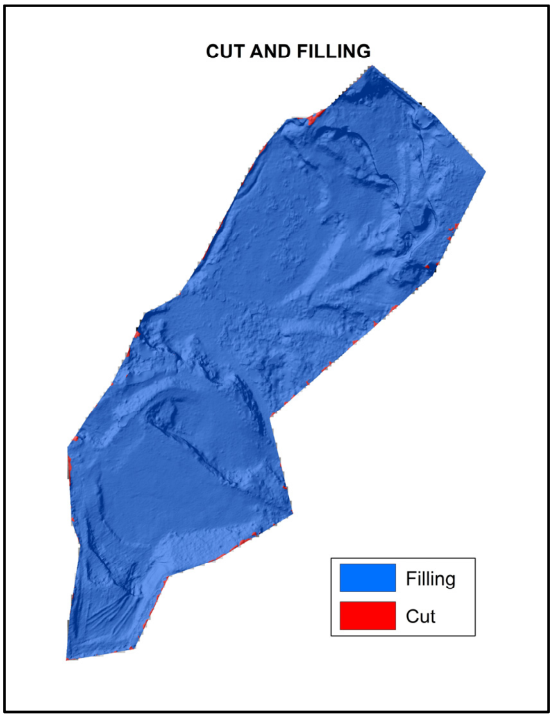

Figure S3. Classification of raster in cut and filling categories.
